# The gingival health status of 8–12 year-old children in Damascus city in Syria during the Syrian Crisis: a cross-sectional epidemiological oral health survey

**DOI:** 10.1186/s13104-018-3998-x

**Published:** 2018-12-13

**Authors:** Muhammed Al-Huda Ballouk, Mayssoon Dashash

**Affiliations:** 1 Paediatric Dentistry Department, Faculty of Dentistry, Damascus University, Damascus, Syria; 2 Centre for Measurement & Evaluation in Higher Education, Ministry of Higher Education, Damascus, Syria

**Keywords:** Gingivitis, Prevalence, Oral health, Plaque Index, Gingival Index, Children, Syria

## Abstract

**Objective:**

The aim of this study was to assess the current gingival health status in children aged 8–12 years in Damascus city. No contemporary data was available with an immense need for studies evaluating the gingival health status in Syrian children, especially under the current circumstances of the Syrian crisis.

**Results:**

The study followed the school-based health survey model using stratified random cluster sampling. A total of 1500 children were clinically examined. The mean PI was at (1.39 ± 0.57) and the mean GI was at (1.12 ± 0.46). Of the total sample, (97.93%) had gingivitis. There is a statistically significant relationship between the used indices means and the children’s distribution as to the city’s localities. Also, there is a statistically significant relationship between the used indices means and the children’s gender. Gingival health was seen to be better and plaque accumulations were less in females. The gingival health status is moderate and is within the acceptable ranges while the plaque accumulations are right above moderate. Still, gingivitis prevalence is high and special care as well as developing and implementing advanced preventive programmes are highly needed.

## Introduction

Oral health is considered as a very important indicator that can be employed to indicate the general health status for persons [1]. It also positively adds up to the person’s physical mental and social health [2, 3]. A good oral health during childhood contributes to a better oral health as an adult later on [4, 5]. Knowing the current oral health status in one society has a big role as well as impact on any therapeutic or preventive interventions which can be adopted on a large scale whether in the educational curricula and teaching strategies or even in oral health awareness programmes [6, 7]. It is also known that the oral health well-being in children is both an important issue and a high responsibility for the society as a whole [8, 9]. A good oral health in one person means mainly the good status of the teeth, gingiva and the supporting periodontal tissues in this person [10].

Therefore, gingival health is a fundamental important aspect of the person’s general health. The presence of gingival diseases is a great indicator for the later development of periodontal diseases and many other oral health related diseases that cause pain and sometimes impairment, and this is besides being the second cause for most teeth losses worldwide [10]. The aim of this research was to assess the current status of the gingival health in children aged 8–12 years in Damascus city to help in enabling health-care professionals improve the oral health status amongst the whole population firstly and secondly in encouraging the adoption of better strategies for delivering the best population-based preventive programmes.

## Main text

### Methods

#### Study design and approach

The study was a cross-sectional epidemiological school-based oral health survey and was conducted in Damascus city in Syria. The field practical part of the study was conducted from September 2016 to January 2017. The study design and methods were based on the World Health Organisation (WHO) guidelines published as the “Oral Health Surveys; Basic Methods” in its 5th edition [11]. Damascus city was divided, as to the health sectors map taken from the Ministry of Health, into nine localities (Al-Zahera and Midan sector, the Old City sector, Al-Mazzeh sector, Al-Muhajirin sector, Dummar and its surroundings sector, Kafarsoseh sector, Barzeh and Rekn Al-Dien sector, Kossor and Kassaa sector, Malki and Abu Rumaneh sector).

#### The study sample and sampling method

The sample size was estimated at a precision level of (0.05) to be (1278). Still, the precision level was raised to (0.03) to increase the study’s quality and reliability making the sample size reach (1444) children. The number was rounded up to (1500) for easier calculations. The confidence interval was at (95%). The multi-stage cluster sampling method was used to select the sample objects. The study sample was comprised of (1500) children of which (745) were females (49.7%) and (755) were males (50.3%).

#### The examination procedure

As expected, carrying out such sort of oral-health examination under crisis circumstances for 1500 children is a tedious job indeed. About 20 children as an average were examined per day. More than 65 working sessions (days) were needed to fulfil the practical field part of the study. All children were examined by a single examiner (MAB). Personal information, Plaque Index of Silness and Loe (PI), and Gingival Index of Loe and Silness (GI) were recorded as a whole and in details for each tooth as well, all according to the WHO criteria. In addition, the participating children were taught how to brush their teeth well and given oral hygiene instructions to improve their overall oral health. Contact was made with the parents/carers of those children in need for treatments and they were referred to the nearest dental office or dental care centre. The diagnostic kit used for the examination included oral mirrors, dental explorers, dental tweezers, and periodontal probes. Also, a head-held light and plaque predisposing tablets were used.

#### Statistical processing

The raw data was processed using IBM SPSS Statistics version 23. Descriptive statistics study was done to reach the mean values for the used indices. Moreover, ANOVA test was implemented to study the relationships between the means of the indices and the distribution according to the localities. Also, T-test was implemented to study the relationships between the means of the indices and the children’s gender.

### Results

Table 1 shows the means for the PI and the GI along with the total children counts for each locality. The means are shown with their standard deviations, and the total means for the whole city are also there. *The mean PI* for the whole city was (1.39 ± 0.57). The highest PI mean in the localities was seen in Al-Zahera and Midan sector reaching (1.58 ± 0.66) while the lowest was recorded in Kossor and Kassaa sector and it reached (1.21 ± 0.52). *The mean GI* for the whole city was (1.12 ± 0.46). The highest GI mean in the localities was again seen in Al-Zahera and Midan sector reaching (1.35 ± 0.57) while the lowest was recorded in Kafarsoseh sector and it reached (0.96 ± 0.37).

**Table 1:** The PI and GI means in the sample children according to their distribution as to the localities and the statistical study for these means in relationship to this distribution

Sector	Children’s count	PI means	*P*-value* for the PI	GI means	*P*-value* for the GI
Zahera and Midan	194	1.588 ± 0.668	*0.000*	1.353 ± 0.573	*0.000*
Old city	155	1.374 ± 0.573		1.201 ± 0.506	
Al-Mazzeh	159	1.542 ± 0.610		1.217 ± 0.486	
Al-Muhajirin	150	1.393 ± 0.667		1.143 ± 0.675	
Dummar	197	1.477 ± 0.499		1.073 ± 0.314	
Kafarsoseh	150	1.271 ± 0.486		0.964 ± 0.370	
Barzeh	153	1.332 ± 0.521		1.013 ± 0.337	
Kossor and Kassaa	191	1.218 ± 0.522		1.037 ± 0.384	
Malki	151	1.326 ± 0.483		1.055 ± 0.225	
Total	*1500*	*1.396* ± 0.575		*1.121* ± 0.463	

* ANOVA

Statistically Significant values are in italic

According to the gingival index values (2.07%) had gingivitis-free healthy gingiva [GI 0], while (77.6%) had mild gingivitis [GI 0.1–1.0], (19.13%) had moderate gingivitis [GI 1.1–2.0], and (1.2%) had severe gingivitis [GI 2.1–3.0]. Overall, (97.93%) of the total sample had gingivitis.

### Discussion

#### Possible reasons for findings and justification of area selection

Malnutrition, the increased consumption of carbohydrates and added artificial sugars found in cheap and low-quality foods, having no water fluoridation, the absence of many primary health-care services, the bad financial situation for plenty, and the crisis situation by itself (which further helped in causing all the aforementioned problems), are all factors which have negatively attributed to the oral health situation (gingival health status included) for the whole population. With such factors taking effect, it is extremely important to know the ground situation we are standing on before implementing any large-scale interventions or adopting nationwide strategies. For these reasons Damascus city was chosen as a location for our research. Damascus now has got residents from various other destinations all around Syria. Many of them fled from their home towns to Damascus because of the war, which makes Damascus a city that has got the full spectrum of the Syrian population not only for being the capital but also for having somehow better situations. Therefore, we can say that our conclusions and results are truly representative of the children’s gingival health in Damascus and can be a good indicator as well for the children’s gingival situation in Syria generally speaking.

#### Geo-demographical comparisons

In the present study, the mean PI was (1.39) which is greater than those PI means shown by other researchers in other populations like in: Damascus (1.12) (in 1996 by Dashash, age group 6–12 years) [12], Jordan (0.61) [13], Sanaa city in Yemen (1.25) [14], Dhamar city in Yemen (1.37) [15], and the Khartoum state in the Sudan (1.30) [16]. In the present study, the mean GI was (1.12) which is greater than other GI means shown by other researchers in different literature of other populations like in: Jordan (0.77) [13], Dhamar city in Yemen (0.98) [15], and the Khartoum state in the Sudan (1.05) [16]. Also, the GI mean reached in our study is smaller than the results shown by other researchers in other populations like in: Damascus (1.51) (again in 1996 by Dashash, age group 6–12 years) [12], Sanaa city in Yemen (1.36) [14], and Mayanamar (1.22) [17]. Also, gingivitis prevalence rate was as high as (97.93%) which is higher than other gingivitis prevalence rates shown in different populations like in: Damascus (90.0%) (again in 1996 by Dashash, age group 6–12 years) [12], Jordan (70.2%) (mild 38.5%, moderate 31.4%, severe 0.3%) [13], Dhamar city in Yemen (86.4%) (mild 59.3%, moderate 25.8%, severe 1.4%) [15], Puerto Rico (80.41%) [18]. Still, the gingivitis prevalence rate reached in this present study was less than that reached in Sanaa city in Yemen in which all the study sample had gingivitis [14].

Possible reasons for such results aside from the current previously mentioned circumstances could be the lack of knowledge regarding the necessity and importance of caring for children’s oral health or may be even due to the parents’ ignorance. Also, it can be the result of the bad financial situation leaving no ability for the families to cover routine preventive visits and measures or even the basics required for a good oral hygiene practice for the children.

#### Relationships outcomes by indices

Tables 1 and 2 summarise all the statistical work regarding the relationships in addition to the indices means. Table 1 shows the statistical significance studies for the PI and the GI in relationship to the children’s distribution as to the localities. The *P*-values for both the PI and the GI statistical studies were 0.000 and therefore they are statistically significant for being < 0.05 (actually very significant, for they are even < 0.01). So, it can be concluded that there is an assessed relationship between the children’s distribution as to the localities and both the PI and the GI means.

**Table 2:** The PI and GI means in the sample children according to their gender and the statistical study for these means in relationship to the children’s gender

PI and GI means in relationship to the children’s gender			
Gender count	Females	Males	*P*-value**
	n = 745	n = 755	
Mean PI	1.346 ± 0.564	1.440015 ± 0.581	*0.001*
Mean GI	1.086± 0.430	1.156± 0.491	*0.004*

** T-test

Statistically Significant values are in italic

Table 2 shows the statistical significance study for the PI and the GI in relationship to the children’s gender. The *P*-value for the PI statistical study was 0.001 and therefore it is statistically significant for being < 0.05 (very significant, < 0.01). So, it can be concluded that there is an assessed relationship between the children’s gender and the PI. The PI mean for males was significantly higher than the PI mean for females. In other words, plaque accumulations were seen to be more in males rather than females. The *P*-value for the GI statistical study was 0.004 and therefore it is statistically significant for being < 0.05 (again very significant, < 0.01). So, it can be concluded that there is an assessed relationship between the children’s gender and the GI. The GI mean for males was significantly higher than the GI mean for females. In other words, the gingiva was seen to be healthier in females rather than males.

All participating children were given thorough advice on the best ways to care for their “smiles” and keep good oral health. These good oral hygiene instructions were passed to the children individually when examining each child and also in groups with the help of visual aids and illustrations. Teachers and school supervisors were also requested to pay good attention to the students’ health and hygiene generally and to the oral health in specific.

### Conclusions

Despite the fact that the gingivitis prevalence was high in the study population, both the PI and the GI were within reasonable moderate ranges and indicate that the gingival health is in a moderately good state. Hence, we can say that the oral health status as to its gingival aspect is still alright, but there is also a room for improvement that can be achieved through comprehensive preventive programmes as well as increasing the awareness about oral health generally and gingival health in specific. Better gingival health status and less plaque accumulations were found in females rather than in their male counterparts.

## Limitations

*Chronological limitations* The practical field part of the study was conducted in 2017 and 2016. Such studies are a continuous need and therefore it is highly advisable that this study is repeated within a period of 3–4 years. *Spatial limitations* The study was undertaken in Damascus and there is a necessity for carrying out studies like this present study in other Syrian cities. *Logical limitations* It is worth mentioning that no test–retest was carried out and this can be considered a limitation for the study.

## Abbreviations

GI: Gingival Index
MAB: Muhammed Al-Huda Ballouk
MD: Mayssoon Dashash
PI: Plaque Index
WHO: World Health Organisation
